# Biomechanical Forces Regulate Gene Transcription During Stretch-Mediated Growth of Mammalian Neurons

**DOI:** 10.3389/fnins.2020.600136

**Published:** 2020-12-08

**Authors:** Joseph R. Loverde, Rosa E. Tolentino, Patricia Soteropoulos, Bryan J. Pfister

**Affiliations:** ^1^Department of Biomedical Engineering, Center for Injury Biomechanics, Materials, and Medicine, New Jersey Institute of Technology, Newark, NJ, United States; ^2^Department of Microbiology, Biochemistry and Molecular Genetics, Genomics Center, Rutgers New Jersey Medical School, Rutgers University, Newark, NJ, United States

**Keywords:** axon stretch growth, mechanotransduction, nervous system development, towed growth, neuron elongation

## Abstract

At birth, there are 100 billion neurons in the human brain, with functional neural circuits extending through the spine to the epidermis of the feet and toes. Following birth, limbs and vertebrae continue to grow by several orders of magnitude, forcing established axons to grow by up to 200 cm in length without motile growth cones. The leading regulatory paradigm suggests that biomechanical expansion of mitotic tissue exerts tensile force on integrated nervous tissue, which synchronizes ongoing growth of spanning axons. Here, we identify unique transcriptional changes in embryonic rat DRG and cortical neurons while the corresponding axons undergo physiological levels of controlled mechanical stretch *in vitro*. Using bioreactors containing cultured neurons, we recapitulated the peak biomechanical increase in embryonic rat crown-rump-length. Biologically paired sham and “stretch-grown” DRG neurons spanned 4.6- and 17.2-mm in length following static or stretch-induced growth conditions, respectively, which was associated with 456 significant changes in gene transcription identified by genome-wide cDNA microarrays. Eight significant genes found in DRG were cross-validated in stretch-grown cortical neurons by qRT-PCR, which included upregulation of *Gpat3, Crem, Hmox1, Hpse, Mt1a, Nefm*, *Sprr1b*, and downregulation of *Nrep.* The results herein establish a link between biomechanics and gene transcription in mammalian neurons, which elucidates the mechanism underlying long-term growth of axons, and provides a basis for new research in therapeutic axon regeneration.

## Significance Statement

This work establishes a link between biomechanics and gene transcription in the developmental growth of mammalian neurons. While links have been established between biomechanics and gene transcription in bone and muscle tissue, a link between physiological axon stretch and gene transcription has not been clearly established in nervous tissue. The method, data, and analysis presented herein provide a conceptual advance in our fundamental understanding of axon growth after growth cone differentiation, whereby a dedicated “stretch-growth” mechanism accommodates for axonal elongation in response to the stimulus of biomechanical stretch. The identified transcriptional changes are poised to spur innovative research toward the regeneration of long axons within the spinal cord and peripheral nervous system.

## Introduction

Individual neurons span remarkable distance through peripheral nerves and white matter tracts, constituting the largest known mammalian cells. Pyramidal neurons of the motor cortex span the order of 50 centimeters through the lateral corticospinal tract, while dorsal root ganglia (DRG) neurons span the order of 200 cm from the toes to the brainstem. The initial growth of the corresponding axons is known to occur via growth cone, which results in the formation of synaptic connections, neuromuscular junctions, and differentiated end-organ receptors. However, over the course of the perinatal period, and throughout childhood, short axons with established synaptic targets continue to grow in synchrony with systemic growth of the enlarging body. This secondary “elongating” phase of axonal growth occurs in the absence of a motile growth cone, through unknown molecular mechanisms.

Intuitively, the expansion of nervous tissue must be synchronized with the expansion of connective tissue to accommodate for the growth of limbs and organs, including expansion caused by pregnancy during adulthood. The emerging regulatory paradigm suggests that axons undergo biomechanical stretch as mitotic tissues naturally expand between neuronal somata and their distal axon terminals. While stretch is distinct from growth, the condition of stretch is known to lead to elongating growth of axons in both peripheral (Bray, 1984; Dennerll et al., 1989; Zheng et al., 1991; Simpson et al., 2013) and central nervous system neurons (Chada et al., 1997). Remarkably, the technique of axon stretch-growth (ASG) has emerged as the sole method for the reproduction of 10 cm-long axon tracts *in vitro* and no upper limit has been identified (Pfister et al., 2004, 2006). Thus, the biomechanical interaction between expanding mitotic tissues and spanning axons is likely to include specific mechanotransduced signals that regulate concomitant growth of neurons.

The evolutionary role of a neuronal “stretch-growth” function is to preserve the integrity of established axons and synaptic connections formed by growth cones. Anecdotally, the role of mechanical force in neurodevelopment has also been implicated in gyrification of the cerebral cortex (Striedter et al., 2015; Garcia et al., 2018) and the displacement and inversion of the hippocampus due to differential growth rates of brain tissues (Thompson et al., 2013). Without the ability to adapt to biomechanical forces, established axons may disconnect from their synaptic targets as tissues naturally expand or change shape throughout the course of life (De Filippo and Atala, 2002).

Outside of the nervous system, biomechanical forces are known to regulate gene expression (see Chan et al., 2010 for review), and to stimulate the growth of bone and muscle (Lammerding et al., 2004; Martin, 2007). However, while it is clear that there is a link between biomechanical stretch and neuron growth, there have been no direct studies of the associated signaling pathways. Here, we recapitulate physiological stretch of isolated embryonic rat neurons using computer-controlled bioreactors. Following 8 days of cumulative stretch-induced growth, RNA was isolated from DRG and cortical neurons, and analyzed by genome-wide cDNA microarray and qRT-PCR arrays, respectively. The results herein establish that there is a link between physiological force and gene transcription in mammalian neurons, which advances fundamental knowledge in neurodevelopment, and provides a basis for new research in therapeutic axon regeneration.

## Materials and Methods

### Stretch-Growth of DRG Neurons

**FIGURE 1:**
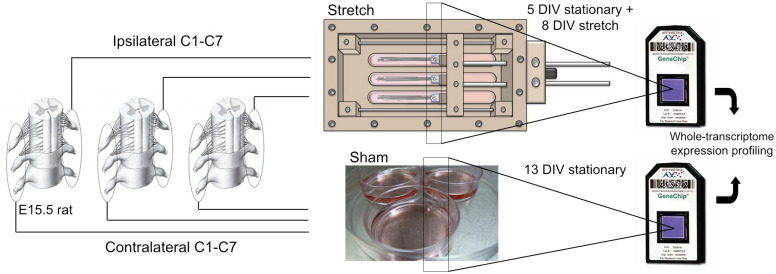
Stretch-growth and DNA microarray analysis of DRG neurons. Biologically paired cervical DRG explants from E15.5 rat pups were seeded onto Aclar manipulating substrates adhered within petri dishes (Sham) and stretch-growth bioreactors (Stretch); see Figure A1 for detail of stretch-growth bioreactor.

**FIGURE A1:**
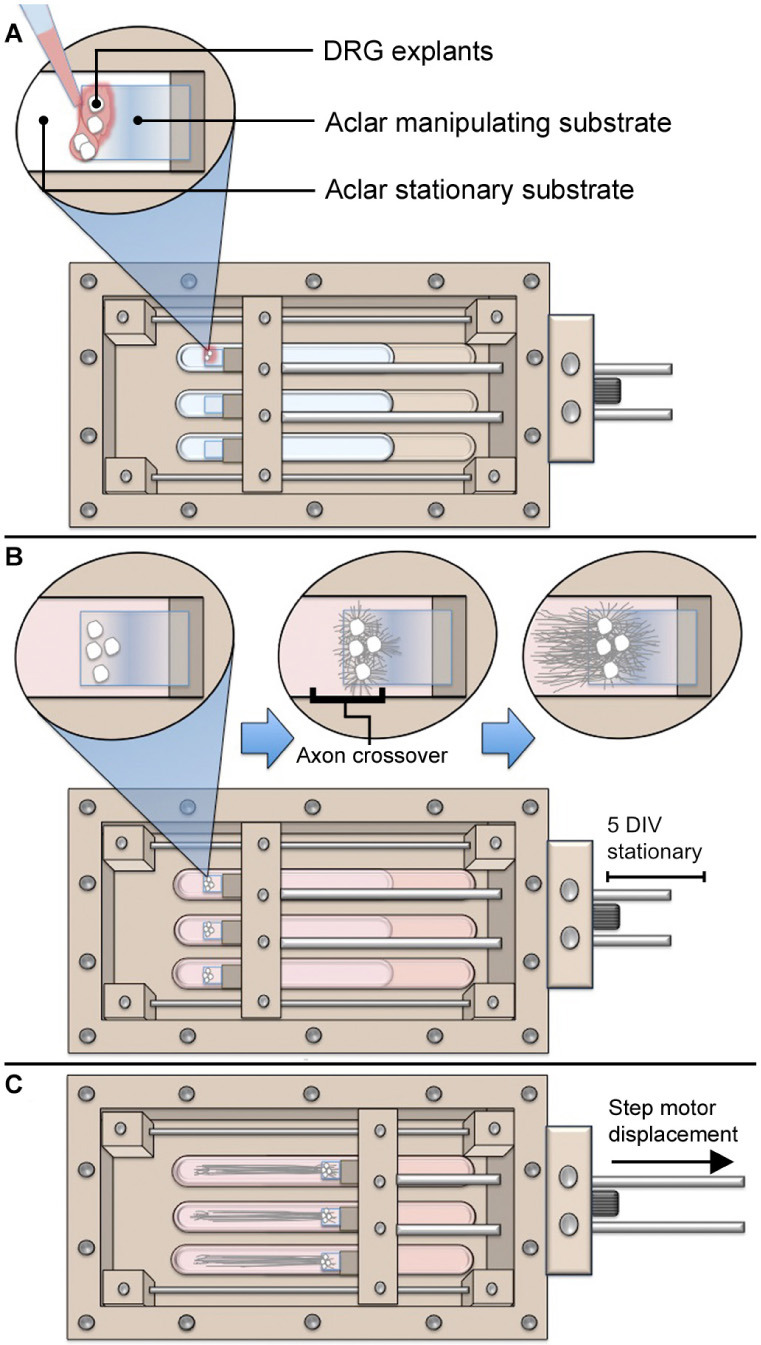
Overview of axon stretch-growth bioreactor usage with DRG. **(A)** DRG explants are seeded at the edges of Aclar manipulating substrates using stereomicroscopy. **(B)** Manipulating substrates are held stationary for 5 days while growth cones extend onto the underlying stationary substrate. **(C)** Spanning axons undergo stretch as the manipulating substrates are displaced in periodic and cumulative 2 μm increments.

Stretch-growth of neuronal cultures was performed using customized bioreactors previously described (Loverde et al., 2011a). For a detailed overview of how stretch-growth is performed, the reader is referred to Loverde et al. (2011b). For gene expression studies, five biologically paired sham and stretch-growth replicates were prepared. For each of five replicates, C1-C7 cervical DRGs were bilaterally isolated from embryonic day 15.5 (E15.5) rat embryos removed from a single timed-pregnant *Sprague-Dawley* rat, and seeded into biologically paired stretch and sham cultures, (Figure 1). Using stereo microscopy, explants were individually seeded adjacent the edge of Aclar manipulating substrates to maximize the outgrowth of all explants onto an underlying Aclar stationary substrate, (Figure A1). Sham cultures consisted of manipulating and stationary substrates glued into 35 mm petri dishes which were seeded identically to bioreactors.

**FIGURE 2:**
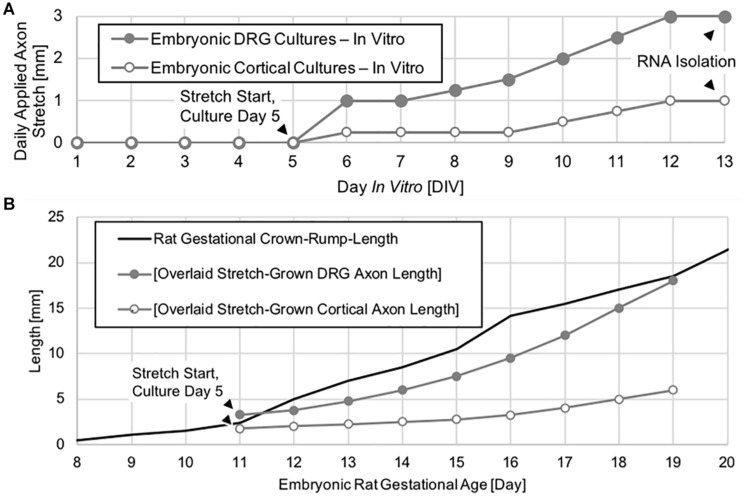
Axon stretch-growth rate escalation paradigm and axon length. **(A)** Neuronal cultures were given 5 days to extend by growth cone under static conditions prior to the initiation of exogenous stretch. Daily stretch was applied in 2 μm increments evenly spaced over 24 h each day. **(B)** DRG neurons underwent stretch-growth at rates corresponding to the peak physiological increase in embryonic rat crown-rump-length occurring between embryonic days 11–19, whereas cortical neurons were stretch grown at a lower rate over the same time period.

Dorsal root ganglia explants were allowed to grow for 5 days by growth cone from the manipulating substrates onto the stationary substrates under static growth conditions. After 5 days, spanning axons were mechanically lengthened for eight consecutive days in cumulative 2 μm increments previously established to be non-injurious (Loverde and Pfister, 2015). By adjusting the delay between increments, DRG neurons were ramped from an elongation rate of 1 to 3 mm/day, corresponding to the peak increase in embryonic rat crown-rump-length (CRL) that occurs between embryonic day 11 and 19, (as adapted from Windhorst and Johansson (1999) and https://embryology.med.unsw.edu.au/), (Figure 2).

### Stretch-Growth of Cortical Neurons

Dissociated cortical neurons were seeded and stretch-grown similar to DRGs. However, cortical neurons were seeded onto both manipulating and stationary substrates to maximize the number of stretch-grown axons. In total, three paired replicates consisting of 6.7 × 10^4^ cells per culture were prepared. Culture sizes were restricted to minimize the presence of non-stretch-grown neurons which could interfere with subsequent analysis of transcriptional change due to stretch. After 5 days of static growth, cortical neurons were ramped from an elongation rate of 0.25 to 1 mm/day, (Figure 2).

### Maintenance of Neuronal Cultures

Culture media consisted of Neurobasal (Gibco 21103) supplemented with 2% B-27 (Gibco 17504), 0.5 mM L-glutamine (Gibco 25030081), while DRGs were further supplemented with 2.5 g/L D-glucose (Sigma G-7528), 1% heat-inactivated FBS, 20 ng/mL NGF (Gibco 13290-010), 20 μM FdU (Sigma F-0503), and 20 μM Uridine (Sigma U-3003), which was changed the day after seeding and every 3 to 4 days thereafter. Once stretch-growth was initiated, media changes took place inside the incubator without interruption of the stretch-growth process.

### Microscopic Analysis of Cultures

Following a total of 13 DIV, cultures were briefly assessed by phase contrast microscopy prior to RNA isolation. Cultures were visually assessed for axon density and integrity, as well as presence of glia. Stretch-grown cultures consisting of low-density axons were excluded from analysis, along with the corresponding biological shams. Similarly, any sham cultures with adhesion inconsistencies or growth abnormalities were grounds for exclusion of the corresponding biologically paired stretch-grown culture. We also confirmed that DRG cultures were devoid of glia prior to RNA isolation to assure the evaluated mRNA was neuronal.

### RNA Isolation

Total RNA was isolated by detaching the Aclar substrates containing somata and immersing directly into microcentrifuges tubes containing lysis buffer. Utilizing a standard silica-based RNA isolation kit (RNeasy, Qiagen, Valencia CA), DNA was digested on-column and eluted RNA was frozen at −80°C. Importantly, stretch-grown cultures underwent continuous stretch until immediately prior to RNA isolation to maintain transcription due to stretch.

### cDNA Microarray Analysis

For DRGs, total RNA was concentrated by speed vacuum to reach ≥100 ng/μL as measured by UV spectrophotometry. Gene expression profiling was performed using 10 Affymetrix GeneChip Rat Gene 1.0 ST Arrays. The array platform interrogates 27,596 genome-wide whole-transcripts using 792,454 distinct well-annotated probes with an average of 29 probes per gene (excluding controls). cDNA was generated from total RNA using the Ambion WT Expression Kit according to the manufacturer’s protocol. The cDNA was fragmented and end labeled following the GeneChip Whole Transcript Terminal Labeling and Hybridization protocol and hybridized to the arrays for 16 h at 45°C in the GeneChip Hybridization Oven 640. Washing and staining with streptavidin-phycoerythrin was performed using the GeneChip Hybridization, Wash, and Stain Kit, and the GeneChip Fluidics Station 450. Chips were scanned using the Affymetrix 3000 7G Plus scanner and the GeneChip Command Console software.

### cDNA Microarray Statistical and Bioinformatics Analysis

Partek Genomics Suite Analysis Software (Partek Inc., St. Louis, MO, United States) was used to analyze microarray data. The arrays were normalized using the robust multi-array average (RMA) algorithm and principal component analysis was performed to check for chip outliers. Pairwise and average fold-changes (FC_P_ and FC_AVG_) were calculated and a paired two-tailed *t*-test was performed to generate *p*-values. Up and downregulation of genes in stretch-grown samples relative to shams were expressed as positive and negative changes, respectively. Significance Analysis of Microarrays (SAM) v4.0 was used to generate *q*-values (Tusher et al., 2001). Normalized data from Partek was analyzed pairwise using SAM to generate *q*-values for all genes. For bioinformatics analysis, significant genes were determined as having an average fold-change (|FC_AVG_|) ≥ 1.2 and *q* < 0.05.

Ingenuity Pathway Analysis (IPA) software (Ingenuity^®^ Systems, Redwood City, CA, United States) was used to study gene expression changes and build interaction pathways. Utilizing IPA’s core analysis function, significant changes in molecular and cellular functions of stretch grown neurons were identified using all data sources, species, tissues, cell lines and mutations from experimentally observed data sources only.

### Quantitative RT-PCR Analysis

Following the DRG gene expression analysis by cDNA microarray, 93 transcripts were chosen for validation by qRT-PCR. For both DRGs and cortical neurons, cDNA was produced and amplified utilizing a custom RT^2^ PreAMP cDNA Synthesis Kit (Qiagen CPBR-12610). Following amplification, custom Qiagen RT^2^ Profiler PCR Arrays (Qiagen CAPR-12610) were run on an Agilent Mx3000P qPCR System. Data was normalized to the housekeeping genes Beta-Actin (*Actb*) and Succinate Dehydrogenase (*Sdha*) and converted to average fold-change using an Excel template (SABiosciences). For DRGs, *P*-values were calculated based on a paired two-tailed *t*-test of the replicate 2^(-Δ Ct) values for each gene in the sham and stretch-grown groups, while equal variance two-tailed *t*-tests were performed for corticals. Significant genes were determined as having |FC_AVG_| ≥ 1.2 and *p* < 0.05.

## Results – DRG Neurons

### Stretch-Growth and Microscopy of DRG Neurons

**FIGURE 3:**
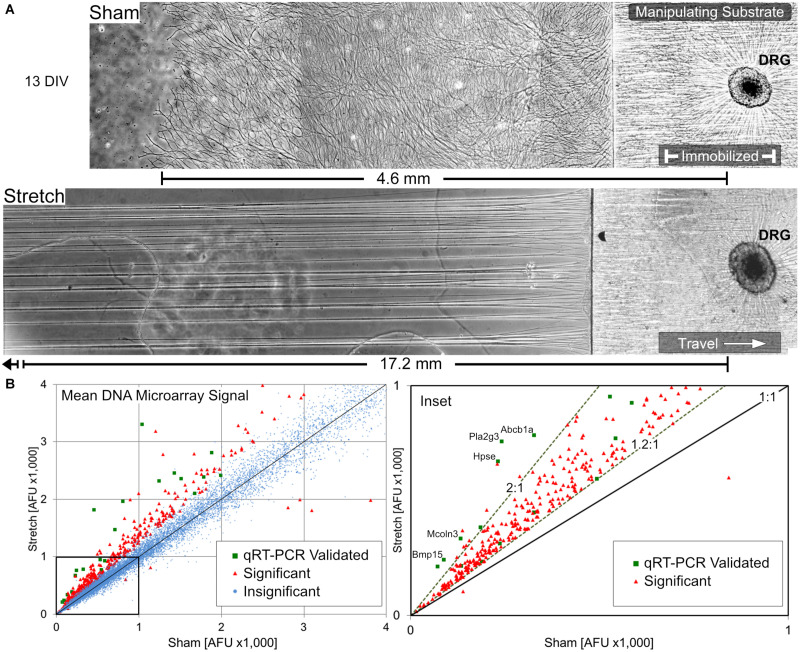
Overview of stretch-growth and DNA microarray results in DRG neurons. **(A)** Representative growth of individual sham and stretch-grown explants after 13 days *in vitro* prior to RNA isolation, see Figure A2 for full panorama. **(B)** Whole-transcriptome analysis with Affymetrix GeneChip Rat Gene DNA Microarrays revealed broad upregulation in stretch-grown DRG. Significant fold-changes were determined by a normalized average microarray fluorescence ratio ≥+1.2:1 or ≤–1.2:1 in stretch-grown cultures relative to sham. Quantitative RT-PCR was used to validate gene expression across the fluorescence spectra of microarray results.

**FIGURE A2:**
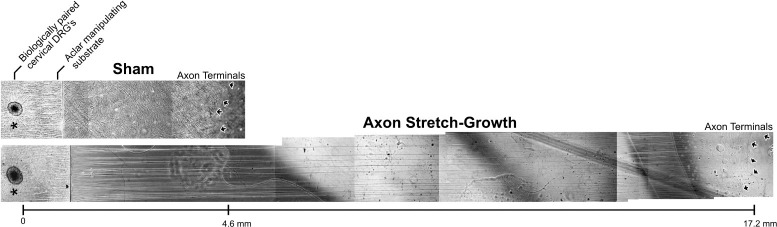
Representative DRG growth at the time of RNA isolation. Biologically paired ipsi- and contralateral cervical DRG explants from a single E15.5 rat pup were cultured for 13 DIV statically (sham) or within bioreactors (axon stretch-growth). While only a single explant is shown for each condition, five explants were seeded per Aclar manipulating substrate adjacent one another, with equivalent growth from each explant verified by phase contrast microscopy. Sham cultures were created by adhering Aclar manipulating and underlying stationary substrates into 35 mm petri dishes, replicating the substrates used in bioreactors. ^∗^Indicates original DRG seeding location atop Aclar manipulating substrates; Arrows indicate the final location of the axon terminals following 13 days in culture.

Following a total of 13 days *in vitro* (DIV) (5 days growth cone extension +8 days stretch or sham condition), cultures were briefly assessed by phase contrast microscopy prior to RNA isolation. Representative growth from biologically paired sham and stretch-grown DRGs are shown in Figure 3A, where the corresponding axons spanned 4.6- and 17.2-mm, respectively (Figure A2 for full panoramic view). In total, 72 sham and 71 paired stretch-grown cervical DRG explants were selected for analysis across five experimental and five control cDNA microarray chips, yielding an average of 14 explants analyzed per chip.

### Statistically Significant Changes in Gene Transcription

456 significant changes in gene transcription were identified in stretch-grown DRG neurons by genome-wide cDNA microarray compared to sham (average fold-change |FC_AVG_| ≥ 1.2 and *q* < 0.05), corresponding to 2% of the total transcripts analyzed, (Figure 3B). Interestingly, 98.6% of significant changes were upregulated (449↑, 7↓), with a range of −2.0 to +5.0-fold. Remarkably, 99.2% of significant findings were unanimously up- or down-regulated across all five biological replicates using the chosen cutoffs and false-discovery statistical approach. For qRT-PCR validation, β-actin (*Actb)* and succinate dehydrogenase (*Sdha*) were chosen as housekeeping genes and had average fold-changes of 1.00 and 1.03, respectively, as determined by DNA microarray. Utilizing 96-well qRT-PCR arrays, 37 significant and 29 insignificant cDNA microarray findings were subsequently validated as statistically significant and insignificant, respectively (|FC_AVG_| ≥ 1.2 and *p* < 0.05 significance threshold for both assays).

### Largest cDNA Microarray Changes – Regulation of Regenerative Associated Genes

The largest fold-changes found by cDNA microarray were three regenerative associated genes (RAGs), including the small proline rich protein-1a (*Sprr1a*, ↑5.0-fold), activating transcription factor-3 (*Atf3*, ↑3.2) and group-III secreted phospholipase-A_2_ (*Pla2g3*, ↑3.2), (Table 1). In studies involving axotomy, and when overexpressed, all three genes are known to increase axonal growth. For an overview on *Sprr1a* the reader is directed to Starkey et al. (2009), for *Atf3* see Hunt et al. (2012), and for *Pla2g3* see Masuda et al. (2008). While *Pla2g3* has normal physiological roles in lipid metabolism and axon myelination (Titsworth et al., 2007), roles for *Sprr1a* and *Atf3* have not been established in the context of normal neuronal function. The results herein suggest that physiological stretch may transiently regulate the expression of specific RAGs during axon elongation, which are known to promote growth and yet are not expressed in mature nervous tissue.

**TABLE 1:** Transcriptional changes in stretch-grown DRG neurons.

			*cDNA Microarray*		*qRT-PCR*	
Symbol^1^	Gene Name	Accession	FC_AVG_^2^	*P*-Value^3^	FC_AVG_^2^	*P*-Value^3^
*Abcb1a*	ATP-binding cassette, sub-family B, member 1A	NM_133401	2.4	**	4.2	**
*Aqp1*	Aquaporin 1	NM_012778	2.7	*	ND	
*Atf3*	Activating transcription factor 3	NM_012912	3.2	****	3.8	*
*Bmp15*	Bone morphogenetic protein 15	NM_021670	2.4	*	11.8	*
*Ccl6*	C-C motif chemokine ligand 6	NM_001004202	−2.0	*	ND	
*Ccne1*	Cyclin E1	NM_001100821	1.2	*	1.2	*
*Chrna1*	Cholinergic receptor, nicotinic, alpha 1	NM_024485	2.1	**	ND	
*Crem*	CAMP responsive element modulator	NM_001110860	1.3	**	2.1	**
*Derl3*	Derlin 3	NM_001109577	2.3	**	ND	
*Dok5*	Docking protein 5	NM_001109344	2.2	*	ND	
*Eif2ak4*	Eukaryotic translation initiation factor 2	NM_001105744	1.6	***	1.7	**
*Fam46a*	Family with sequence similarity 46 member A	NM_001106844	2.1	**	ND	
*Gadd45a*	Growth arrest and DNA-damage-inducible alpha	NM_024127	1.9	*	3.1	**
*Got1*	Glutamic-oxaloacetic transaminase 1	NM_012571	1.2	***	1.3	*
*Gpat3*	Glycerol-3-Phosphate Acyltransferase 3	NM_001025670	2.1	**	2.2	*
*Gzmb*	Granzyme B	NM_138517	2.4	***	9.6	*
*Hk2*	Hexokinase 2	NM_012735	2.8	*	4.0	*
*Hmox1*	Heme oxygenase (decycling) 1	NM_012580	2.6	**	4.0	***
*Hpse*	Heparanase	NM_022605	3.0	***	4.3	**
*Hsp90b1*	heat shock protein 90 kDa	NM_001012197	1.3	*	1.5	*
*Hspa5*	Heat shock protein family A member 5 (70 kDa)	NM_013083	1.4	**	2.1	***
*Hspb1*	Heat shock protein 1 (Hsp 27 kDa)	NM_031970	2.1	*	3.8	*
*Hspb8*	heat shock protein B8	NM_053612	1.3	**	1.3	*
*Ier3*	Immediate early response 3	NM_212505	1.5	*	2.4	*
*Insig2*	Insulin induced gene 2	NM_178091	1.6	**	ND	
*Kdm3a*	Lysine (K)-specific demethylase 3A	NM_175764	1.5	*	1.4	*
*Klf6*	Kruppel-like factor 6	NM_031642	1.3	**	1.2	*
*Lpin1*	Lipin 1	NM_001012111	1.7	**	ND	
*Mcoln3*	Mucolipin 3	NM_001012059	2.5	**	5.1	**
*Mmp12*	Matrix metallopeptidase 12	NM_053963	2.9	*	ND	
*Mpz*	Myelin protein zero	NM_017027	−2.0	*	−2.3	*
*Mt1a*	Metallothionein 1a	NM_138826	1.7	*	2.0	*
*Nefh*	Neurofilament, heavy polypeptide	NM_012607	1.3	**	2.2	*
*Nefm*	Neurofilament, medium polypeptide	NM_017029	1.1	*	1.5	**
*Notum*	Notum pectinacetylesterase homolog	NM_001013975	2.1	****	ND	
*Nrep*	Neuronal regeneration related protein (P311)	NM_178096	−1.4	***	−2.1	**
*Nrg1*	Neuregulin 1	NM_031588	1.2	*	ND	
*Nupr1*	Nuclear protein, transcriptional regulator, 1	NM_053611	1.9	**	2.3	*
*P4ha1*	Prolyl 4-hydroxylase, alpha polypeptide I	NM_172062	1.5	**	1.6	**
*Pcdh21*	Protocadherin 21	NM_053572	2.0	**	ND	
*Pfkfb4*	Phosphofructokinase/Fructose bisphosphatase	NM_019333	2.1	**	2.3	***
*Pgm2*	Phosphoglucomutase 2	NM_001106007	2.2	**	ND	
*Pla2g3*	Phospholipase A2, group III	NM_001106015	3.2	***	22.0	*
*Pparg*	Peroxisome proliferator-activated receptor	NM_001145367	1.4	*	2.7	0.150
*Rras*	Harvey rat sarcoma virus oncogene	NM_001108481	1.5	**	ND	
*Rsad2*	Radical S-adenosyl methionine domain cont. 2	NM_138881	2.6	**	3.1	0.077
*Serpinb2*	Serine (or cysteine) peptidase inhibitor	NM_021696	2.2	*	ND	
*Slc2a1*	Solute carrier family 2, member 1	NM_138827	1.8	*	4.2	**
*Slc2a3*	Solute carrier family 2, member 3	NM_017102	1.3	*	1.7	**
*Sprr1a*	Small proline-rich protein 1A	NM_021864	5.0	**	13.0	**
*Sprr1b*	Small proline-rich protein 1B	XM_001065728	1.2	***	18.5	*
*Tgfb3*	Transforming growth factor, beta 3	NM_013174	1.6	****	1.5	*
*Thy1*	Thy-1 cell surface antigen	NM_012673	1.1	**	1.4	*
*Tlcd1*	TLC domain containing 1	NM_001013858	2.1	**	ND	
*Tspyl2*	TSPY-like 2	NM_001191618	1.2	**	1.8	**

^1^List includes all significant cDNA microarray changes ≥ 2.0-fold, qRT-PCR validated changes, and curated findings. All listed genes were ubiquitously up- or down-regulated across five biologically paired replicates for each assay. ^2^Average Fold Change: Average expression of stretch-grown replicates relative to shams. ^3^Paired two-tailed t-test: (*) P < 0.05; (**) P < 0.01; (***) P < 0.001; (****) P < 0.0001.

In comparing the remainder of significant cDNA microarray findings against reported RAGs from literature, we found upregulation in 8 out of 40 known RAGs evaluated in total (Costigan et al., 2002; Schmitt et al., 2003; McPhail et al., 2004; Hanz and Fainzilber, 2006; Ben-Yaakov and Fainzilber, 2009; Huebner and Strittmatter, 2009; Richardson et al., 2009; Michaelevski et al., 2010; Zigmond, 2011). Additional RAG upregulation was also found for *Hk2, Mt1a, Il6*, and *Hspa5* (from highest to least fold-change), while downregulation was found for *Nrep*. Significant changes for the remainder of the evaluated RAGs were not found, such as the transcription factors *C-Jun* and *Stat3*, the cytoskeletal proteins *Gap43/Cap23*, α7-integrin, nor the translation regulators *Mtor* or *Pten*, (Table A1). We conclude that stretch-mediated growth upregulates a subset of the stress-response genes previously identified through studies of axonal injury, but that the scope of transcriptional change is distinct from trauma.

### Upregulation of the Adaptive Cellular Stress Response

Utilizing the Ingenuity Pathway Analysis (IPA) core analysis tool, significant microarray data was compared with experimentally derived data in published literature to predict changes in cell function and phenotype. In stretch-grown DRGs, IPA discovered a significant increase in cell viability and decreased cell death, which was based on evidence in literature from neural, epithelial, testicular, and pancreatic tissues, (Table 2). Our data significantly overlapped with the canonical unfolded protein response (*p* < 0.01) and endoplasmic reticulum stress response (*p* < 0.05) pathways, which are associated with the adaptive cellular stress response. Notable and upregulated genes included heat shock protein-5 (*Hspa5*, ↑1.4), which is an ER-localized RAG found within lesioned axons (Willis et al., 2005), together with *Hsp90b1* and the *Hsp40* chaperones (*Dnajc3*, ↑1.4 & *Dnajb9*, ↑1.3) produced during ER stress (Kurisu et al., 2003). We also found upregulation of *Gadd45a*, which is mutually upregulated in DRGs following trauma, and is involved in excision of damaged DNA (Costigan et al., 2002).

**TABLE 2:** Bioinformatics analysis of DRG neurons.

Disease or Function^1^	Z-Score^2^	Genes^3^	*P*-Value^4^
**Lipid Metabolism**			
Metabolism of membrane lipid			
derivative	2.9	22	**
Metabolism of phospholipid	2.4	15	**
Uptake of fatty acid	2.4	7	**
Transport of fatty acid	2.1	7	**
Synthesis of lipid	2.0	39	**
Accumulation of fat	2.0	4	***
Metabolism of acylglycerol	2.0	11	**

**Cell Survival**			
Cell viability	5.6	66	***
Cell survival	5.6	72	***
Necrosis	−2.0	121	***
Cell death	−2.1	145	***
Cell death of pancreas	−2.6	8	*
Necrosis of epithelial tissue	−2.6	33	**

**Organismal Survival**			
Survival of organism	2.9	41	***
Discomfort	2.0	19	**
Failure of kidney	−2.2	17	***
Edema	−2.4	21	*
Organ degeneration	−2.4	30	**
Oxidative stress	−2.6	8	***

**Cellular Organization**			
Organization of cytoskeleton	3.5	65	**
Microtubule dynamics	3.3	56	**
Cellular homeostasis	2.7	77	***
Formation of cell protrusions	2.2	45	**

**Neurological Injury**			
Damage of cortical neurons	−2.2	5	****
Damage of cerebral cortex	−2.4	6	***
Damage of nervous system	−2.4	20	****
Damage of brain	−2.6	17	***

^1^Findings determined by Ingenuity Pathway Analysis software. ^2^Predicted activation state; < 0: decreased, > 0: increased. ^3^Number of significant genes overlapping with each function. ^4^Right-tailed Fisher’s exact test: (*) P < 0.01; (**) P < 0.005; (***) P < 0.001; (****) P < 0.0001.

**FIGURE 4:**
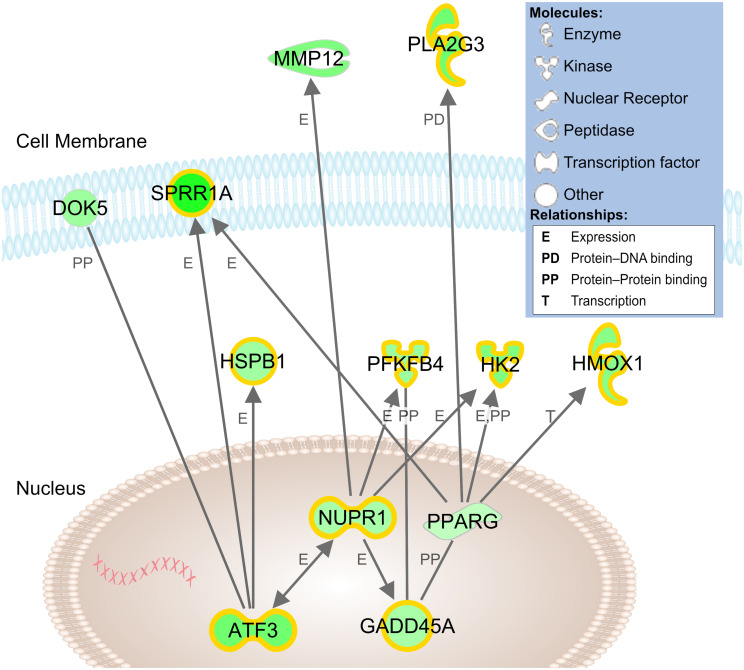
Network model of cDNA microarray findings in stretch-grown DRG neurons. Connections between genes reflect relationships established in literature and identified by Ingenuity Pathway Analysis software. Green shading reflects level of upregulation of stretch-grown samples relative to sham; highlighting reflects significant qRT-PCR validated findings. Gene locations are approximate based of expected subcellular localization established in literature.

In our network analysis of microarray findings, the most interconnected gene among the highest fold changes was the stress-responsive transcription factor *Atf3* (Figure 4). In addition to promoting growth, *Atf3* is known to inhibit Jun-mediated apoptosis through upregulation of heat shock protein 27 kDa (*Hspb1*, ↑2.1) and downstream activation of Akt (Lewis et al., 1999; Nakagomi et al., 2003). Similarly, a number of lower-fold transcription factors with dual roles in axon growth and stress signaling were upregulated, including *Crem*, *Klf6*, and *Ccne1*, (Table 1).

While *Atf3* is known as a broad cellular stress sensor, we also found upregulation of a major downstream target, the transcription factor nuclear protein-1 (*Nupr1*, ↑1.9). While *Nupr1* has not been previously associated with neuron growth, *Nupr1* serves as a hub for downstream metabolic stress signaling. In our network analysis, *Nupr1* served as a connecting node between *Atf3* and the glycolytic enzymes hexokinase-2 (*Hk2*, ↑2.8) and phosphofructokinase (*Pfkfb4*, ↑2.2), which regulate energy production (Panasyuk et al., 2012). *Nupr1* also works in concert with the peroxisome proliferator-activated receptor-γ (*Pparg*, ↑1.4), a key regulator of glucose homeostasis and lipid metabolism. We conclude that physiological stretch appears to initiate an adaptive cellular stress response, which may inhibit apoptosis and increase cellular metabolism during biomechanical stretch.

### Changes in Membrane Lipid Production and Composition

Categorically, the largest number of changes in our IPA core analysis occurred in the functional area of lipid metabolism, which included increases in membrane lipid, phospholipid, and acylglycerol metabolism, (Table 2). Our data significantly overlapped with the CDP-diacylglycerol, triacylglycerol, and phosphatidylglycerol (PG) biosynthesis canonical pathways (*p* < 0.001), which were all predicted as upregulated (*z* > 2.0). Notably, PG is a ubiquitous membrane phospholipid, and a key intermediate in the synthesis of other lipids. Phosphatidylglycerol is also hydrolyzed by *Pla2g3* in the production of lysophospholipids, which can affect cell shape and increase membrane fluidity (Lundbaek and Andersen, 1994). In this capacity, we also note upregulation of the water channel protein aquaporin-1 (*Aqp1*, ↑2.7), which increases the passive permeability of the membrane to water. Finally, we note the upregulation of glycerol-3-phosphate acyltransferase-3 *(Gpat3*, ↑2.2), which catalyzes production of the bioactive phospholipid lysophosphatidic acid (LPA) known to modulate neurodevelopment (Frisca et al., 2012).

**FIGURE 5:**
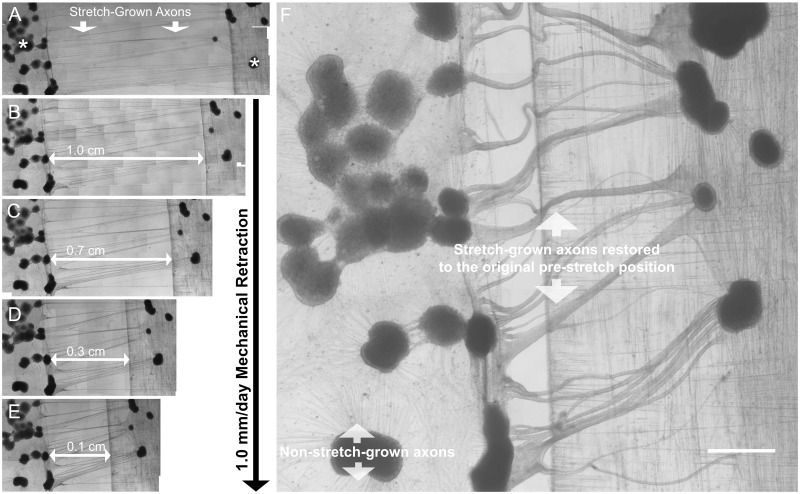
Morphology of stretch-grown axons following retraction. **(A)** In retraction assays, axons were initially stretch-grown to 1.3 cm in length over 13 days; asterisks indicate DRG explant locations following stretch-growth. **(B–E)** Axons were relieved of all applied stretch over 13 days at a rate of –1.0 mm/day. **(F)** At the original pre-stretch position, enlargement and undulations are evident in previously stretch-grown axons. Non-stretch-grown axons visible within the same culture appear taught and of normal diameter; Bar = 1 mm.

In parallel studies, we found that stretch-grown axons become enlarged and undulated when returned to the length preceding stretch. Following stretch-growth at a constant rate of 1.0 mm/day for 13 days, and “retraction” at –1.0 mm/day for 13 days, the diameters of axons which had undergone stretch-growth were grossly enlarged and undulated compared to non-stretch-grown controls, (Figure 5), suggesting the addition of mass due to stretch. Critically, despite stretch-growth at a rate comparable to growth cone extension, stretch resulted in enlargement along the entirety of stretch-grown axons rather than localized changes near the hillock or tip. Given the necessity for the axonal membrane to continue to grow following growth cone differentiation, these results suggest a relationship between the composition of the membrane and its state of biomechanical stretch.

Interestingly, we also found upregulation of genes associated with myelination, such as *Insig2* (Leblanc et al., 2005), and *Lpin* (Verheijen et al., 2003), but marked downregulation of myelin protein zero (*Mpz*, ↓2.0), which is typically produced by Schwann Cells. Significant changes of other myelin proteins were not found, however, there was mild upregulation of neuregulin (*Nrg1*, ↑1.2) in every biological replicate.

### Cytoskeletal Reorganization

In the category of cellular organization, IPA predicted increases in cytoskeletal organization, microtubule dynamics, and cellular homeostasis. In our network analysis we found upregulation of *Hspb1* and docking protein-5 (*Dok5*, ↑2.2), both of which interact with *Atf3* and are also involved in reorganization of the growth cone cytoskeleton (Grimm et al., 2001; Sun et al., 2013). Related lower-fold changes included upregulation of the second messenger Rho family GTPase *RhoQ* (↑1.3), the RAS-related protein *Rras* (↑1.5), Plexin-B1 (↑1.5), and Semaphorin-6A (↑1.4), which all have roles in growth cone initiation and guidance (Hall and Lalli, 2010). While the specific functions of these genes are unclear in the context of stretch-growth, the discovered findings suggest an increase in actin-cytoskeletal remodeling coinciding with stretch. We theorize that cytoskeletal reorganization may be necessary to accommodate for integration of new materials, such as membrane lipids, microfilaments, and structural proteins, which may ultimately allow for extrinsic elongation and relief of applied tension.

## Results – Cortical Neurons

### Stretch-Growth and Microscopy of Cortical Neurons

**FIGURE 6:**
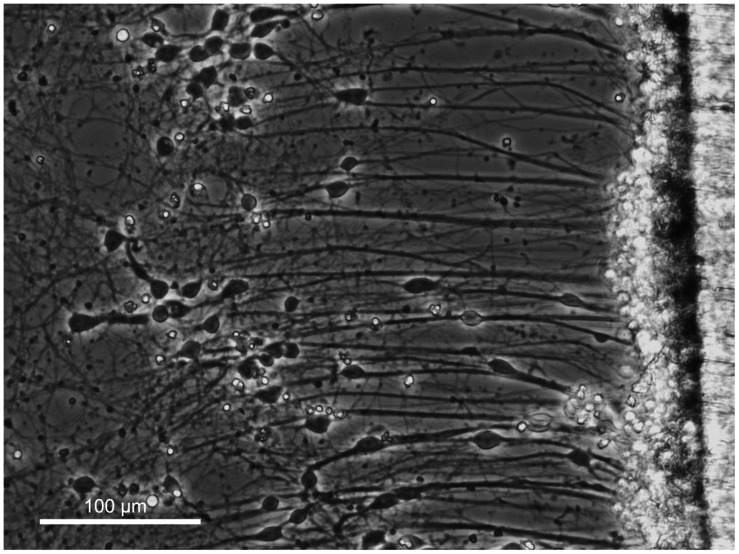
Axon stretch-growth of cortical neurons. Dissociated cortical neurons were seeded at the interface of a stationary and motile towing substrate (far right) to maximize the number of stretch-grown axons. Culture sizes were restricted to minimize the presence of non-stretch-grown neurons which could interfere with analysis of stretch-growth gene expression. Bar = 100 μm.

Similar to DRGs, dissociated cortical neurons were seeded into biologically paired stretch and sham cultures for a total of 13 DIV. At the time of RNA isolation, stretch-grown cortical neurons only spanned 6 mm due to a reduced stretch rate as compared to DRGs. At equivalent stretch rates, axon breakage occurred in cortical cultures, and so we reduced the rate of stretch until the density and integrity of stretch-grown cortical axons, (Figure 6), matched the normal appearance of stretch-grown DRG axons. While DRGs were ramped from an elongation rate of 1 to 3 mm/day, cortical neurons were only ramped from 0.25 to 1 mm/day, (Figure 2). In total, 2 × 10^5^ stretch-grown and 2 × 10^5^ sham cortical neurons were selected for analysis across three experimental and three control qRT-PCR arrays.

### Statistically Significant Changes in Gene Transcription

18 significant gene expression changes were identified in stretch-grown cortical neurons utilizing the same 96-well qRT-PCR arrays and housekeeping genes chosen to validate our DRG microarray findings, (Table 3). Similar to DRGs, the majority of significant changes in stretch-grown cortical neurons were upregulated (12↑, 6↓). We also found that 8 out of 18 significant cortical gene expression changes overlapped with significant DRG findings, suggesting there is mechanistic overlap in the stretch-growth of both peripheral and central nervous system neurons.

**TABLE 3:** Transcriptional changes in stretch-grown cortical neurons.

			*qRT-PCR*	
Symbol	Gene Name	Accession	FC_*AVG*_^1^	*P*-Value^2^
*Acta1*	Actin, alpha 1, skeletal muscle	NM_019212	−1.4	*
*Adm*	Adrenomedullin	NM_012715	3.9	**
*Ar*	Androgen receptor	NM_012502	1.8	**
*Crem*	CAMP responsive element modulator	NM_001110860	2.8	**
*Gap43*	Growth associated protein 43	NM_017195	−1.3	**
*Gpat3*	Glycerol-3-Phosphate Acyltransferase 3	NM_001025670	1.9	*
*Hmox1*	Heme oxygenase (decycling) 1	NM_012580	2.4	*
*Hpse*	Heparanase	NM_022605	1.6	*
*Hrk*	Harakiri, BCL2 interacting protein (BH3 domain)	NM_057130	−1.6	*
*Mt1a*	Metallothionein 1a	NM_138826	8.8	*
*Nampt*	Nicotinamide phosphoribosyltransferase	NM_177928	1.5	*
*Nefm*	Neurofilament, medium polypeptide	NM_017029	1.6	*
*Nkap*	NFKB activating protein	NM_001024872	−1.2	*
*Nrep*	Neuronal regeneration related protein	NM_178096	−1.3	*
*Pten*	Phosphatase and tensin homolog	NM_031606	1.4	**
*Sprr1b*	Small proline-rich protein 1B (cornifin)	XM_001065728	2.2	*
*Stat3*	Signal transducer and activator of transcription 3	NM_012747	1.9	****
*Stom*	Stomatin	NM_001011965	−1.5	**

^1^Average Fold Change: Average expression of three stretch-grown replicates relative to biologically paired shams.^2^Two-sample equal variance two-tailed t-test: (*) P < 0.05; (**) P < 0.01; (***) P < 0.001; (****) P < 0.0001.

### Common Transcriptional Changes in Stretch-Grown DRG and Cortical Neurons

**FIGURE 7:**
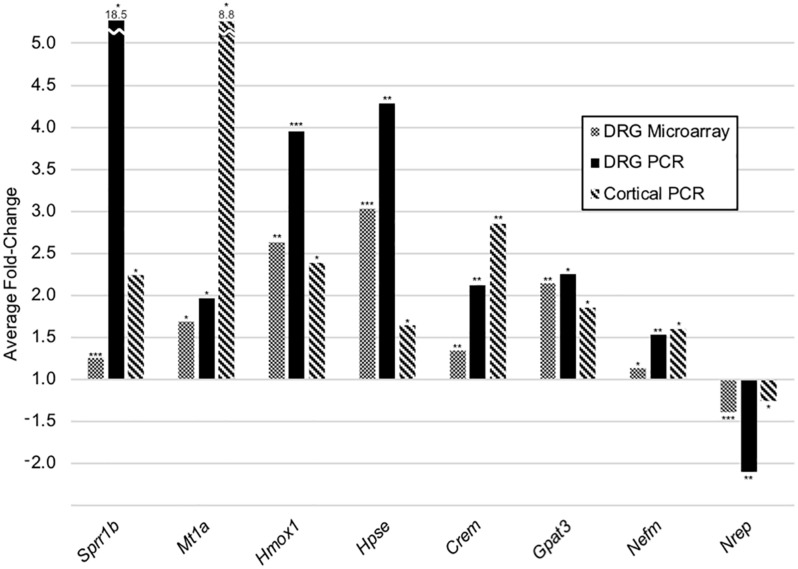
Common transcriptional changes in stretch-grown DRG and cortical neurons. Listed genes were significant across three independent assays, including five replicate DRG cDNA microarrays, five replicate DRG qRT-PCR arrays, as well as three replicate cortical qRT-PCR arrays. All DRG stretch–sham replicates were evaluated using paired two-tailed *t*-tests, while cortical neurons were evaluated using two-sample equal variance two-tailed *t*-tests: (*) *P* < 0.05, (**) *P* < 0.01, (***) *P* < 0.001.

Despite dissimilar stretch-growth rates and final axon lengths, eight genes were mutually significant across our DRG and cortical neuron assays, including upregulation of *Sprr1b, Mt1a, Hmox1, Hpse, Crem, Gpat3, Nefm*, and downregulation of *Nrep* (in order of decreasing combined average fold-change), (Figure 7). Briefly, metallothionein (*Mt1a)* is known to be involved in metal ion homeostasis, while heme oxygenase *(Hmox1)* is an enzyme involved in heme catabolism. Interestingly, while both *Mt1a* and *Hmox1* serve as antioxidants, their transcription was disproportionally greater in corticals and DRGs, respectively. Two additional enzymes were mutually upregulated, including heparanase (*Hpse)*, which cleaves heparin sulfate proteoglycans in ECM remodeling, and glycerol-3-phosphate acyltransferase-3 *(Gpat3)*, which catalyzes production of LPA. Finally, we note mild upregulation of neurofilament medium chain (*Nefm*), together with downregulation of neuronal regeneration related protein (*Nrep*). Mechanistically, *Nefm* is associated with maintenance of axon caliber, while *Nrep* is associated with the MECP2 pathway and Rett Syndrome, and is known to promote axonal regeneration. Interestingly, the regulation of both *Nefm* and *Nrep* were opposite their typical expression following injury (Fernandes et al., 1999).

The upregulation of *Sprr1a* in DRGs and *Sprr1b* in DRGs and cortical neurons is remarkable. Small proline-rich (SPR1) proteins are normally crosslinked in the formation of an insoluble layer underlying the membrane of stratified epithelia (Steinert et al., 1998; Candi et al., 1999). SPR1 and SPR2 proteins are known to increase following UV irradiation of epithelial cells (Kartasova and van de Putte, 1988), while *Sprr1a* and *Sprr2a* act as cardio-protective agents during biomechanical stress and ischemia in cardiac tissue (Pradervand et al., 2004). It has been postulated that SPR proteins are cytoprotective and stabilize the structure of the cytoskeleton and myofibrils during stress in keratinocytes and cardiomyocytes, respectively. *Sprr1a* has also been postulated to modulate actin dynamics in the growth cones of regenerating DRGs (Bonilla et al., 2002). These findings suggest that SPR proteins are responding to biomechanical stimuli in neurons, and may act to temporarily stabilize the axonal cytoskeleton during stretch.

Finally, we note the mutual upregulation of the cAMP responsive element modulator (*Crem*), a basic leucine zipper (bZIP) transcription factor that binds the cAMP responsive element (CRE) promoter. While bZIP factors are known to regulate processes such as cell survival and lipid metabolism, Crem is also known to modulate activity-dependent synaptogenesis and plasticity in neurodevelopment (Aguado et al., 2009). Complementarily, Atf3 is a cAMP responsive element-binding (CREB) protein, which may act in concert with Crem. While *Atf3* was among the largest changes in DRG neurons, it was insignificantly upregulated in stretch-grown cortical neurons, perhaps due to the difference in stretch-growth rate between DRG and cortical neurons in the assays performed herein.

## Discussion

The seminal works of Paul Weiss, Dennis Bray, and Steve Heidemann established that axons maintain a basal resting tension, and actively grow to relieve applied stretch. In a process involving hundreds of transcriptional changes, we found that physiological stretch promotes increases in cell viability, lipid metabolism, and cytoskeletal reorganization in DRG neurons. ER stress resulting from stretch appears to drive an adaptive cellular stress response involving transcription factors (*Atf3*, *Crem*, *Klf6*, *Ccne1*) historically associated with both stress and neuron growth. The ensuing growth component of the response is composed of changes that modulate membrane metabolism and integrity (*Pla2g3, Gpat3, Sprr1a*), actin cytoskeletal remodeling (*Hspb1, Dok5, Rras*), and changes that decrease extracellular adhesion (*Mmp12, Hpse*). Through the stretch-growth process, the condition of stretch may be gradually relieved as axons remodel and integrate new materials to morphologically assume extrinsic changes in length.

We theorize that the transcription and expression of stretch-growth genes intuitively varies with organismal growth. For example, the crown-rump-length of a human fetus increases at a rate of 1 mm/day in the first trimester, but periodically accelerates to 2 mm/day in the second trimester (Aviram et al., 2004). In rats, the crown-rump-length can increase by up to 3 mm/day, however, during slower periods of development, biomechanical signaling is likely attenuated and expectedly quiescent through most of adulthood. We conclude that stretch-responsive genes are likely to be expressed transiently to meet the demands of body growth, which must be taken into consideration in future *in vivo* research.

In our genome-wide cDNA microarray analysis, the amplitude of transcriptional changes were relatively low, with a maximum fold-change of 5.0 corresponding to the stress-responsive *Sprr1a.* Strikingly, the reported increase for *Sprr1a* ranges up to 3,119-fold as found by qRT-PCR (Seijffers et al., 2007) in neuronal injury studies, suggesting that the stimulus of developmental stretch is relatively mild. Since stretch-growth expectedly occurs over prolonged time periods and at relatively low amplitude, we conclude that small changes in gene expression are a characteristic of healthy stretch-growth, despite there may be variability corresponding to the transient level of stretch.

Research by Bonilla, Tanabe, and Seijffers suggests that overexpression of *Sprr1a* or *Atf3* leads to enhanced axon outgrowth, while knockout of either retards growth. Seijffers et al. also demonstrated that *Sprr1a* expression is higher in injured mice than in *Atf3* overexpressing mice, suggesting that *Sprr1a* is not solely regulated by *Atf3*. Indeed, following injury, *Sprr1a* is regulated by many factors, including the regenerative genes *Jun* and *Cebpb*. In a study of cardioprotective pathways, Sprr1a was found to be downstream of the gp130 pathway, which implies potential activation by a large number of pro-inflammatory cytokines and transcription factors (Pradervand et al., 2004). However, at the time of this writing, the only significant genes known to regulate *Sprr1a* during stretch-growth were *Atf3* and *Pparg*. Such differences between injury and physiological stretch may account for the difference in amplitude of *Sprr1a* transcription, together with the expression of other RAGs, and serve to differentiate the process of stretch-growth from growth-cone extension and regeneration.

## Data Availability Statement

The cDNA microarray data from this study has been deposited in NCBI’s Gene Expression Omnibus (Edgar et al., 2002) and are accessible through GEO Series accession number GSE98535 (https://www.ncbi.nlm.nih.gov/geo/query/acc.cgi?acc=GSE98535).

## Ethics Statement

All animal protocols were approved by the Rutgers University IACUC.

## Author Contributions

JL and BP: conceptualization. JL, PS, and BP: methodology and writing – review and editing. JL and PS: data curation and formal analysis. JL, RT, and PS: investigation. PS and BP: resources and supervision. JL: writing – original draft and visualization. BP: funding acquisition.

## Conflict of Interest

The authors declare that the research was conducted in the absence of any commercial or financial relationships that could be construed as a potential conflict of interest.

## Appendix

**TABLE A1:** Regenerative associated gene transcription in stretch-grown DRGs.

			*cDNA Microarray*		*qRT-PCR*	
Symbol	Gene Name	Accession	FC_AVG_^1^	*P*-Value^2^	FC_AVG_^1^	*P*-Value^2^
*Arg1*	Arginase 1	NM_017134	−1.0	0.892	1.1	0.790
*Atf3*	Activating transcription factor 3	NM_012912	3.2	****	3.8	*
*Basp1*	Brain abundant, attached signal protein 1 (Cap23)	NM_022300	−1.0	0.489	1.5	0.160
*Bdnf*	Brain derived neurotrophic factor	NM_012513	1.3	0.105	2.7	0.239
*Braf*	V-raf murine sarcoma viral oncogene homolog B1	AF352172	1.0	0.610	ND	
*Cebpb*	CCAAT/enhancer binding protein beta	NM_024125	1.1	0.466	−1.2	0.362
*Cntf*	Ciliary neurotrophic factor	NM_013166	1.2	0.165	ND	
*Dpysl2*	Dihydropyrimidinase-like 2	NM_001105717	1.0	0.283	ND	
*Fgf2*	Fibroblast growth factor, basic	NM_019305	−1.2	0.492	−1.5	0.300
*Gap43*	Growth associated protein 43	NM_017195	−1.0	0.714	−1.1	0.644
*Gdnf*	Glial derived neurotrophic factor	NM_019139	1.1	0.286	2.7	*
*Gfap*	Glial fibrillary acidic protein	NM_017009	−1.2	0.138	ND	
*Gfra1*	Glial cell line-derived neurotrophic factor receptor	NM_012959	1.0	0.975	1.3	0.134
*Hk2*	Hexokinase 2	NM_012735	2.8	*	4.0	*
*Hspa1b*	Heat shock protein 1 b, 70 kDa	NM_212504	1.0	0.994	1.2	0.432
*Hspa5*	Heat shock protein family A member 5, 70 kDa	NM_013083	1.4	**	2.1	***
*Il6*	Interleukin 6	NM_012589	1.6	**	1.7	0.077
*Itga7*	Integrin-α7	NM_030842	1.0	0.942	2.4	0.304
*Jun*	Jun proto-oncogene	NM_021835	1.0	0.572	1.5	0.331
*Kpnb1*	Karyopherin subunit beta 1 (Importin β1)	NM_017063	1.0	0.799	ND	
*Lif*	Leukemia inhibitory factor	NM_022196	−1.0	0.672	ND	
*Mapk1*	Mitogen activated protein kinase 1 (Erk2)	NM_053842	1.0	0.759	−1.1	0.468
*Mapk12*	Mitogen activated protein kinase 12 (Sapk3)	NM_021746	1.3	*	−1.1	0.182
*Mapk14*	Mitogen activated protein kinase 14 (p38)	NM_031020	1.1	0.064	−1.1	0.759
*Mapk3*	Mitogen activated protein kinase 3 (Erk1)	NM_017347	−1.0	0.591	1.6	0.244
*Mapk8*	Mitogen activated protein kinase 8 (Jnk)	NM_053829	1.1	0.362	−1.0	0.927
*Mt1a*	Metallothionein 1a	NM_138826	1.7	*	2.0	*
*Mtor*	Mechanistic target of rapamycin	NM_019906	−1.0	0.546	−1.0	0.724
*Nfkb1*	Nuclear factor of kappa light polypeptide enhancer	NM_001276711	1.0	0.085	ND	
*Ngf*	Nerve growth factor	NM_001277055	1.1	0.262	1.3	0.103
*Nrep*	Neuronal regeneration related protein (P311)	NM_178096	−1.4	***	−2.1	**
*Pla2g3*	Phospholipase A2, group III	NM_001106015	3.2	***	22.0	*
*Pten*	Phosphatase and tensin homolog	NM_031606	1.0	0.361	1.2	0.183
*Raf1*	V-raf-leukemia viral oncogene 1	NM_012639	1.0	0.631	ND	
*Ranbp1*	RAN binding protein 1	NM_001108324	−1.0	0.448	ND	
*Sox11*	SRY-box containing gene 11	NM_053349	1.2	0.243	1.4	*
*Sprr1a*	Small proline-rich protein 1A	NM_021864	5.0	**	13.0	**
*Stat3*	Signal transducer and activator of transcription 3	NM_012747	1.0	0.530	−1.0	0.809
*Trpc4*	Transient receptor potential cation channel	NM_080396	1.0	0.757	ND	
*Vim*	Vimentin	NM_031140	−1.2	0.107	ND	

*^1^Average Fold Change: Average expression of five stretch-grown replicates relative to five biologically paired shams.^2^Paired two-tailed t-test: (*) P < 0.05; (**) P < 0.01; (***) P < 0.001; (****) P < 0.0001.*
